# P15 The main aetiological factors of lung damage in severe pneumonia and sepsis in military servicemen: retrospective analysis for 2023

**DOI:** 10.1093/jacamr/dlae136.019

**Published:** 2024-08-23

**Authors:** Oksana Viltsaniuk, Viktor Syvak, Tatiana Lysak, Nataliia Sokolovska, Martyniuk Viktoria, Oleksandr Viltsaniuk

**Affiliations:** National Pirogov Memorial Medical University, Vinnytsia, Ukraine; Military Medical Clinical Centre of the Central Region, Ukraine; Military Medical Clinical Centre of the Central Region, Ukraine; Military Medical Clinical Centre of the Central Region, Ukraine; Military Medical Clinical Centre of the Central Region, Ukraine; National Pirogov Memorial Medical University, Vinnytsia, Ukraine

## Abstract

**Background:**

Today, the problem of pulmonary pathology is very relevant in Ukraine, as it is not always possible to provide timely and high-quality medical care owing to the Russian invasion of Ukraine.

**Objectives:**

To analyse the types of the main aetiological factors of lung pathology and their susceptibility to different groups of antimicrobial agents used at different stages of treatment of these patients.

**Materials and methods:**

Microbiological studies of 36 case histories of severe patients who were inpatients in the pulmonology department of the Military Medical Centre were analysed.

**Results:**

The main diseases that caused lung pathology were community-acquired pneumonia (44%), septic pneumonia (29%) and nosocomial pneumonia (27%). When studying the extent of lung involvement, the most common was involvement of four and five lobes of the lungs, which accounted for 61% of all cases, and the most common complication was exudative pleurisy (57%). In the course of studying the microbiological characteristics of pathogens, a monoculture was isolated in 80% of cases, in 12% of cases there was a combination of *Staphylococcus aureus* and *Klebsiella pneumoniae*, and in 8% of cases *S. aureus*, *K. pneumoniae* and *Enterobacter* were identified. *S. aureus* and *K. pneumoniae* were most often isolated from sputum and pleural fluid. Susceptibility testing showed that in the vast majority of cases *S. aureus* was susceptible to meropenem, linezolid, cefepime, cefoperazone, ciprofloxacin and amikacin. *K. pneumoniae* was susceptible to cefepime and ciprofloxacin in only 2% of cases, while it was resistant to other antibiotic groups. Pathogens isolated from blood were mostly *S. aureus* and *Staphylococcus haemolyticus*, which were susceptible to meropenem and vancomycin, while resistance to ceftazidime, moxifloxacin, cefazolin and amikacin was noted.

**Conclusions:**

The isolated pathogens are resistant to the main groups of antimicrobial drugs recommended for use according to the AWaRe antibiotic classification in the initial stages of empirical antimicrobial therapy. This is of concern because there is an active use of reserve drugs, which in turn also contributes to the progression of resistance to such drugs. In addition, active migration of people between countries may lead to the spread of this trend beyond Ukraine.

